# Socioeconomic Inequality in mortality using 12-year follow-up data from nationally representative surveys in South Korea

**DOI:** 10.1186/s12939-016-0341-9

**Published:** 2016-03-22

**Authors:** Young-Ho Khang, Hye-Ryun Kim

**Affiliations:** Department of Health Policy and Management, Seoul National University College of Medicine, 103 Daehak-ro Jongno-gu, Seoul, 03080 South Korea; Institute of Health Policy and Management, Seoul National University Medical Research Center, Seoul, South Korea; Korea Institute for Health and Social Affairs, Sejong, South Korea

**Keywords:** Cause of death, Mortality, Republic of Korea, Socioeconomic factors

## Abstract

**Background:**

Investigations into socioeconomic inequalities in mortality have rarely used long-term mortality follow-up data from nationally representative samples in Asian countries. A limited subset of indicators for socioeconomic position was employed in prior studies on socioeconomic inequalities in mortality. We examined socioeconomic inequalities in mortality using follow-up 12-year mortality data from nationally representative samples of South Koreans.

**Methods:**

A total of 10,137 individuals who took part in the 1998 and 2001 Korea National Health and Nutrition Examination Surveys were linked to mortality data from Statistics Korea. Of those individuals, 1,219 (12.1 %) had died as of December 2012. Cox proportional hazard models were used to estimate the relative risks of mortality according to a wide range of socioeconomic position (SEP) indicators after taking into account primary sampling units, stratification, and sample weights.

**Results:**

Our analysis showed strong evidence that individuals with disadvantaged SEP indicators had greater all-cause mortality risks than their counterparts. The magnitude of the association varied according to gender, age group, and specific SEP indicators. Cause-specific analyses using equivalized income quintiles showed that the magnitude of mortality inequalities tended to be greater for cardiovascular disease and external causes than for cancer.

**Conclusion:**

Inequalities in mortality exist in every aspect of SEP indicators, both genders, and age groups, and four broad causes of deaths. The South Korean economic development, previously described as effective in both economic growth and relatively equitable income distribution, should be scrutinized regarding its impact on socioeconomic mortality inequalities. Policy measures to reduce inequalities in mortality should be implemented in South Korea.

**Electronic supplementary material:**

The online version of this article (doi:10.1186/s12939-016-0341-9) contains supplementary material, which is available to authorized users.

## Background

Socioeconomic health inequalities have become an important public health concern worldwide [1, 2]. The examination of socioeconomic inequalities in mortality using nationally representative follow-up data is a crucial step toward a deeper understanding of the nature and magnitude of socioeconomic health inequalities in a country. Several Western countries have data infrastructures including nationally representative surveys, mortality surveillance data, and personal identifiers for the linkage of these two sources of data [3–9].

Studies on socioeconomic mortality inequalities have been carried out in Asian countries [10, 11]. A few studies from Asian countries have employed nationally representative samples [12, 13], but the sample sizes of these studies were relatively small and they involved a relatively short period of mortality follow-up (<5 years), which hindered the identification of age-, gender-, and cause-specific patterns of inequality. Other Asian studies with a longer mortality follow-up period included several survey sites [14–16], but were not nationally representative. With the exception of a few studies including elderly Chinese subjects [17], investigations into socioeconomic inequalities in mortality have rarely used long-term mortality follow-up data from nationally representative samples in Asian countries.

In South Korea, several sources of national mortality follow-up data are available that allow the examination of socioeconomic inequalities in mortality [18–20], but mortality follow-up data from the Korean National Health and Nutrition Examination Surveys (KNHANES) have been an especially valuable source for studies of mortality inequalities [21–24]. Although prior South Korean studies have shown mortality inequalities associated with several indicators of socioeconomic position (SEP) [21, 23, 24], neither gender-specific analyses nor cause-specific analyses have been performed due to the relatively small number of deaths in the population of those studies. Moreover, prior studies from Western countries on socioeconomic inequalities in mortality using nationally representative follow-up data have employed a limited subset of SEP indicators [3–9]. Each SEP indicator has distinct influences in promoting or damaging an individual’s health [25]. SEP indicators cannot be used interchangeably in assessing health inequalities [21, 26]. Use of a wide range of SEP indicators in measuring health inequalities may provide information on relatively important socioeconomic aspects in determining health and/or neglected areas of health inequalities in a society. In this study, we used follow-up 12-year mortality data to examine socioeconomic inequalities in mortality according to a wide range of SEP indicators. We also identified cause-, gender-, and age-specific patterns of mortality inequalities.

## Methods

### Study subjects

Data were obtained from two waves (1998 and 2001) of the KNHANES. A stratified multistage probability sampling method was employed to select study subjects representing the civilian, non-institutionalized population of South Korea. In both 1998 and 2001 KNHANES, 200 primary sampling units for the whole country were drawn for health examination. About twenty households were sampled for each PSU using systematic sampling. In the sampled households, individuals aged 10 or over were invited for health examination. Details regarding the study design, methods, and variables are available elsewhere [24, 27]. The KNHANES in 1998 and 2001 was composed of four surveys: a health interview, a health examination, a health behavior survey, and a nutrition survey. The health examination surveys in 1998 and 2001 collected 13-digit personal identification numbers (PINs) for 11,969 individuals 30 years of age and older. A total of 10,532 men and women reported valid PINs. We excluded 395 subjects whose PINs corresponded to demographic information different from the information obtained in the interviews or who had missing information regarding the SEP parameters used in this study. A total of 10,137 individuals (84.7 % of the original 11,969 subjects) were linked to mortality data from Statistics Korea. Of those individuals, 1,219 (12.1 %) had died as of December 2012 (see Additional file 1: Table S1). Adulthood mortality data in South Korea have been shown to be complete [27, 28].

Since the data used in this study were collected from official national surveys, participant consent on data linkage was not specifically obtained. The data linkage was made by the Korea Institute for Health and Social Affairs, which conducted the 1998 and 2001 KNHANES [27]. We used linked data without personal identifiers for our analyses. This study was approved by the Seoul National University Hospital Institutional Review Board, Seoul, South Korea.

### Indicators of socioeconomic position (SEP)

A wide range of SEP indicators were employed as the independent variables in this study. Education level was determined by the highest level of education that an individual had completed. Individuals were grouped into five categories: no formal education, elementary school, middle school, high school, and college or higher. For cause-, age group-, and gender-specific analyses, we used three categories: elementary school or lower, middle school, and high school or higher. Using information on personal occupational categories for both men and women, we employed two types of occupation-related SEP parameters: occupation and occupational class. Occupations were divided into manual, non-manual, and others. The category of manual occupations included service and sales workers, agricultural and fishery workers, craft and related trade workers, plant and machine operators and assemblers, and elementary occupations, whereas the category of non-manual occupations included managers, professionals, technicians, and clerks. Those who were not in the labor market (unemployed, retired, students, and housewives) were classified in the ‘other’ category. Occupational class was determined using a classification system originally suggested by Hong and colleagues [29], which uses a Korean standard occupational classification and considers employment status (employer, employee, and self-employed) [24]. This system categorizes occupational class as follows: high and middle-high; middle; laborers; agriculture, fishery, and self-employed workers; low social class, and others. We also used the Statistics Korea definition of employment status as an SEP indicator with five categories: those employed in standard employment settings, the self-employed, employers, those employed in non-standard employment settings, and others. In this classification, standard employment settings refer to permanent work (work with a permanent contract), whereas nonstandard employment settings include temporary work (work with a contract period of less than one year) and daily work (work with a contract period of less than one month). Monthly household income was measured as combined income from all sources and family members in the household and grouped into four categories: 3,000 USD or more, 2000–2999 USD, 1000–1999 USD, and less than 1000 USD. We also used equivalized income quintiles, calculated as household income÷ (household size)^0.5^. The type of health insurance participants had was used as SEP. Public servant health insurance, employee health insurance, and self-employed health insurance constituted national health insurance. Those with medical aid program or no health insurance were also identified. The subjects were asked to report household living standards with five answer categories: very rich, rich, fair, poor, or very poor. The ‘rich’ and ‘very rich’ categories were combined considering the low percent of respondents and used as the reference category. In the 2001 KNHANES, variables for monthly living expenditures, home ownership, and housing type were also identified and included as SEP indicators. Monthly living expenditures were measured as combined living expenditures in the household and grouped into five categories: 2,000 USD or more, 1500–1999 USD, 1000–1499 USD, 500–999 USD and less than 500 USD. Information on home ownership was also obtained in the survey. The housing type was determined at the household interview with five categories: apartment, detached house, semidetached house, multiplex house, house in a building for commerce, or others. The housing type was grouped into apartment, house (including all types of house), and other.

### Mortality

The outcome variables in this study were mortality from all causes and from four broad causes of death. The causes of death were identified from death certificates, following the International Classification of Disease, 10th Revision (ICD-10) codes. We classified causes of death into four groups: cancer (ICD-10 codes: C00–C97), cardiovascular diseases (ICD-10 codes: I00–I99), external causes (ICD-10 codes: V01–Y89), and others. The date of death was also obtained from the death certificate data.

### Statistical analysis

All the statistical analyses were performed using SAS version 9.1 (SAS Institute, Cary, NC, USA). Cox proportional hazard models were used to estimate the relative risk (RR) of mortality (hazard ratios with Cox regression were used as an approximation of the RR) associated with SEP indicators, adjusted for survey year (1998 and 2001), gender, and age (both age and age squared were included in the models). Considering that the magnitude of mortality inequalities varies with age and gender [3, 4], age- and gender-specific analyses were conducted. For gender-specific analyses, the gender variable was not adjusted for in the model. All analyses were performed after taking into account primary sampling units, stratification, and sample weights, which makes analysis results representative of the target population. If the p-value is under 0.05, results were considered statistically significant.

## Results

The study cohort contained 122,610 person-years of follow-up (Additional file 1: Table S1). The average follow-up period was 12.1 years. Of 1,219 total deaths, cancer (*n* = 325), cardiovascular disease (*n* = 298), external causes (*n* = 135), and other causes (*n* = 461) accounted for 26.7 %, 24.5 %, 11.1 %, and 37.8 % of deaths, respectively. Additional file 1: Table S1 also shows gender- and age-specific follow-up durations and numbers of deaths, presenting gender- and age- based differences in mortality rates.

Table 1 presents socioeconomic differences measured by RR for all-cause mortality when survey year, gender, age, and age squared were adjusted for and primary sampling units, stratification, and sample weights were taken into account. All SEP indicators were associated with all-cause mortality, and the associations showed a gradient pattern. For example, compared to those with a college or higher level of education, those with no formal education were 2.14 times (95 % confidence interval [CI]: 1.49–3.08) more likely to die during the follow-up period, while those with an elementary, middle, and high school education had 1.66 (95 % CI: 1.19–2.30), 1.59 (95 % CI: 1.15–2.20), and 1.37 (95 % CI: 1.01–1.85) times greater risks of all-cause mortality, respectively. The RR of manual workers was 3.01 (95 % CI: 1.85–4.92) compared with non-manual workers, while those not in the labor market showed a 4.29 (95 % CI: 2.61–7.07) times greater mortality risk. Compared with the high/middle-high occupational class, all other occupational classes showed significantly increased risks of mortality. Both measures for income—monthly household income and equivalized income quintiles—showed gradient patterns of mortality risks among men and women 30 years of age and older. The results of the analysis also showed differences in mortality according to types of health insurance. In addition, a gradient pattern of mortality risks associated with self-rated living standards was also observed. Table 1 also presents that, for most SEP indicators, the magnitude of the relationship with all-cause mortality tended to be greater among adults 30–64 years of age than among the elderly. However, among elderly subjects, increased mortality risks were associated with equivalized income quintiles, types of health insurance, and self-rated living standards.

**Table 1:** Number of subjects, number of deaths, and relative risks (adjusted for age and gender) of mortality from all causes by age group: follow-up 12-year mortality data from the 1998 and 2001 Korea National Health and Nutrition Examination Surveys

	Men and women 30 years of age and older		Men and women 30–64 years of age		Men and women 65 years of age and older	
	Number of subjects (deaths)	RR (95 % CI)	Number of subjects (deaths)	RR (95 % CI)	Number of subjects (deaths)	RR (95 % CI)
Education						
College or higher	1880 (67)	1.00 (reference)	1816 (41)	1.00 (reference)	64 (26)	1.00 (reference)
High school	3389 (191)	1.37 (1.01–1.85)	3258 (142)	1.70 (1.16–2.49)	131 (49)	0.86 (0.52–1.44)
Middle school	1625 (157)	1.59 (1.15–2.20)	1503 (103)	2.02 (1.32–3.11)	122 (54)	1.03 (0.62–1.71)
Elementary school	2031 (344)	1.66 (1.19–2.30)	1570 (169)	2.26 (1.44–3.57)	461 (175)	0.99 (0.63–1.56)
No formal education	1212 (460)	2.14 (1.49–3.08)	424 (51)	2.94 (1.63–5.33)	788 (409)	1.26 (0.79–2.01)
Occupation						
Non-manual	1328 (21)	1.00 (reference)	1308 (16)	1.00 (reference)	20 (5)	1.00 (reference)
Manual	5010 (492)	3.01 (1.85–4.92)	4531 (308)	3.85 (2.25–6.60)	479 (184)	1.41 (0.41–4.91)
Other	3799 (706)	4.29 (2.61–7.07)	2732 (182)	5.15 (2.92–9.10)	1067 (524)	2.25 (0.66–7.75)
Occupational class						
High/middle-high class	1473 (32)	1.00 (reference)	1454 (27)	1.00 (reference)	19 (5)	1.00 (reference)
Middle class	1144 (89)	2.52 (1.64–3.89)	1092 (69)	2.83 (1.77–4.52)	52 (20)	1.68 (0.48–5.82)
Laborers	1467 (71)	2.01 (1.28–3.17)	1442 (65)	2.52 (1.56–4.06)	25 (6)	0.56 (0.13–2.48)
Agricultural/fishery/self-employed	799 (207)	2.15 (1.40–3.32)	553 (92)	2.73 (1.65–4.53)	246 (115)	1.21 (0.38–3.92)
Low social class	1509 (347)	3.08 (2.03–4.68)	1048 (140)	3.99 (2.50–6.34)	461 (207)	1.70 (0.54–5.43)
Other	3745 (473)	3.08 (1.99–4.76)	2982 (113)	3.46 (2.01–5.95)	763 (360)	1.84 (0.57–5.96)
Monthly household income (USD)						
≥3000	925 (47)	1.00 (reference)	862 (20)	1.00 (reference)	63 (27)	1.00 (reference)
2000–2999	1713 (107)	1.27 (0.87–1.84)	1593 (58)	1.48 (0.83–2.64)	120 (49)	1.05 (0.64–1.72)
1000–1999	4019 (325)	1.52 (1.08–2.14)	3690 (184)	1.90 (1.11–3.25)	329 (141)	1.19 (0.76–1.85)
<1000	3480 (740)	1.90 (1.34–2.71)	2426 (244)	2.39 (1.38–4.14)	1054 (496)	1.58 (0.99–2.52)
Equivalized income quintile						
I (highest)	1914 (116)	1.00 (reference)	1770 (58)	1.00 (reference)	144 (58)	1.00 (reference)
II	2128 (131)	1.07 (0.81–1.41)	1993 (72)	0.99 (0.68–1.44)	135 (59)	1.23 (0.80–1.87)
III	1965 (171)	1.27 (0.96–1.67)	1767 (97)	1.50 (1.05–2.16)	198 (74)	1.05 (0.71–1.55)
IV	2137 (298)	1.60 (1.25–2.04)	1809 (144)	1.67 (1.18–2.38)	328 (154)	1.53 (1.09–2.13)
V (lowest)	1993 (503)	1.55 (1.22–1.96)	1232 (135)	1.66 (1.17–2.37)	761 (368)	1.50 (1.09–2.07)
Type of health insurance						
National health insurance	9752 (1097)	1.00 (reference)	8339 (475)	1.00 (reference)	1413 (622)	1.00 (reference)
Medical aid program	315 (108)	1.57 (1.23–2.01)	170 (23)	2.49 (1.53–4.06)	145 (85)	1.36 (1.05–1.77)
No health insurance	70 (14)	2.45 (1.37–4.40)	62 (8)	2.22 (1.01–4.90)	8 (6)	2.94 (1.18–7.32)
Self-rated living standard						
Rich or higher	368 (38)	1.00 (reference)	303 (10)	1.00 (reference)	65 (28)	1.00 (reference)
Fair	5768 (520)	1.03 (0.71–1.48)	5085 (237)	0.95 (0.45–2.01)	683 (283)	1.04 (0.69–1.56)
Poor	3318 (496)	1.31 (0.90–1.90)	2696 (201)	1.17 (0.55–2.52)	622 (295)	1.33 (0.88–2.00)
Very poor	683 (165)	1.72 (1.15–2.56)	487 (58)	1.79 (0.80–3.97)	196 (107)	1.61 (1.03–2.52)

*USD* US dollars; *RR* relative risk; *CI* confidence interval

Additional file 1: Table S2 shows mortality differences associated with the use of different categories of SEP indicators. The use of different categories for education, occupational class, and type of health insurance led to patterns of mortality disparities similar to those presented in Table 1. Additional file 1: Table S2 also presents findings regarding SEP indicators that were not included in Table 1. Compared to workers in standard working settings, non-standard workers had a 2.30 (95 % CI: 1.55–3.41) times greater mortality risk, and the self-employed also had a 1.65 (95 % CI: 1.21–2.24) times greater mortality risk. Additional file 1: Table S2 also showed differences in the magnitude of socioeconomic mortality differences between two broad age groups (35–64 years of age and 65 years of age and older).

Table 2 presents gender-specific RRs of all-cause mortality according to SEP indicators. The magnitude of the relationship with mortality tended to be greater among men than women for occupation, occupational class, monthly household income, and self-rated living standards, while similar or even greater RRs among women than men were found for education, equivalized household income, and type of health insurance. However, no meaningful interactions between gender and SEP indicators were found. For example, the *P*-value for the interaction between gender and education (ordinal variable) was 0.140. *P*-values for the interactions of gender with the manual and other categories of occupation were 0.226 and 0.360, respectively. Additional file 1: Table S3 shows findings reflecting the use of different categories of SEP indicators and other SEP indicators not used in Table 2. No interaction terms between gender and SEP indicators resulted in meaningful gender differences in RRs (all *P*-values >0.05).

**Table 2:** Gender-specific relative risks (adjusted for age) of mortality from all causes: follow-up 12-year mortality data from the 1998 and 2001 Korea National Health and Nutrition Examination Surveys

	Men 30 years of age and older		Women 30 years of age and older	
	Number of subjects (deaths)	RR (95 % CI)	Number of subjects (deaths)	RR (95 % CI)
Education				
College or higher	1189 (58)	1.00 (reference)	691 (9)	1.00 (reference)
High school	1696 (160)	1.37 (0.99–1.89)	1693 (31)	1.21 (0.53–2.79)
Middle school	746 (122)	1.57 (1.11–2.23)	879 (35)	1.69 (0.71–4.04)
Elementary school	805 (228)	1.62 (1.13–2.30)	1226 (116)	1.99 (0.79–4.98)
No formal education	246 (143)	1.93 (1.27–2.96)	966 (317)	2.87 (1.11–7.38)
Occupation				
Non-manual	1002 (18)	1.00 (reference)	326 (3)	1.00 (reference)
Manual	2850 (381)	3.26 (1.92–5.56)	2160 (111)	1.54 (0.45–5.28)
Other	830 (312)	4.73 (2.73–8.21)	2969 (394)	2.25 (0.66–7.66)
Occupational class				
High/middle-high class	1144 (29)	1.00 (reference)	329 (3)	1.00 (reference)
Middle class	818 (75)	2.48 (1.57–3.93)	326 (14)	2.04 (0.55–7.54)
Laborers	938 (60)	2.03 (1.25–3.29)	529 (11)	1.50 (0.39–5.81)
Agricultural/fishery/self-employed	656 (183)	2.29 (1.42–3.68)	143 (24)	1.59 (0.43–5.84)
Low social class	919 (269)	3.42 (2.18–5.36)	590 (78)	2.08 (0.59–7.28)
Other	207 (95)	3.81 (2.28–6.36)	3538 (378)	2.10 (0.62–7.19)
Monthly household income (USD)				
≥3000	433 (29)	1.00 (reference)	492 (18)	1.00 (reference)
2000–2999	811 (63)	1.25 (0.80–1.97)	902 (44)	1.22 (0.64–2.32)
1000–1999	1946 (183)	1.51 (1.01–2.26)	2073 (142)	1.41 (0.79–2.54)
<1000	1492 (436)	2.13 (1.40–3.23)	1988 (304)	1.56 (0.86–2.83)
Equivalized income quintile				
I (highest)	874 (66)	1.00 (reference)	1040 (50)	1.00 (reference)
II	928 (74)	1.04 (0.73–1.49)	1200 (57)	1.17 (0.76–1.81)
III	928 (90)	1.19 (0.84–1.69)	1037 (81)	1.28 (0.81–2.03)
IV	1050 (190)	1.76 (1.29–2.42)	1087 (108)	1.44 (0.98–2.12)
V (lowest)	902 (291)	1.58 (1.16–2.15)	1091 (212)	1.59 (1.09–2.30)
Type of health insurance				
National health insurance	4547 (663)	1.00 (reference)	5205 (434)	1.00 (reference)
Medical aid program	101 (40)	1.29 (0.86–1.93)	214 (68)	1.82 (1.36–2.42)
No health insurance	34 (8)	2.58 (1.14–5.87)	36 (6)	2.43 (1.10–5.40)
Self-rated living standard				
Rich or higher	169 (22)	1.00 (reference)	199 (16)	1.00 (reference)
Fair	2722 (307)	1.05 (0.68–1.62)	3046 (213)	0.93 (0.50–1.73)
Poor	1516 (298)	1.40 (0.89–2.20)	1802 (198)	1.08 (0.58–2.02)
Very poor	275 (84)	1.82 (1.10–3.02)	408 (81)	1.49 (0.78–2.82)

*USD* US dollars; *RR* relative risk; *CI* confidence interval

Table 3 presents mortality risks by SEP indicators for four broad causes of death. Due to the small numbers of cause-specific deaths, combined categories for education and occupational class were used. As the number of deaths from external causes were small in the reference categories, high RRs for mortality from external causes were found to be associated with occupation, monthly household income, and self-rated living standards. Meanwhile, although the number of deaths in non-manual occupations was generally low for the four broad causes of death, the results of our analysis showed increased cause-specific RRs among those in the manual and other occupational categories. When we used equivalized income quintiles to evenly allocate study subjects into each category of SEP, thereby obtaining relatively a large number of deaths in each SEP group, the magnitude of mortality inequalities tended to be greater for cardiovascular and external deaths, while no significant RRs were found for cancer deaths. Increased mortality risks by equivalized income quintile were found for other causes of death. For occupational class, increased mortality risks were found for cardiovascular deaths and deaths from other causes. Interestingly, those with a middle school education showed a greater risk of mortality from cardiovascular diseases, while a weaker relationship was observed in those with an elementary school or lower education.

**Table 3:** Relative risks (adjusted for age and gender) of mortality from four broad causes: follow-up 12-year mortality data from the 1998 and 2001 Korea National Health and Nutrition Examination Surveys

	Number of subjects	Cancer deaths		Cardiovascular deaths		External deaths		Deaths from other causes	
		Number of deaths	RR (95 % CI)	Number of deaths	RR (95 % CI)	Number of deaths	RR (95 % CI)	Number of deaths	RR (95 % CI)
Education									
High school or higher	5269	94	1.00 (reference)	50	1.00 (reference)	42	1.00 (reference)	72	1.00 (reference)
Middle school	1625	46	0.95 (0.64–1.42)	43	1.68 (1.07–2.65)	25	1.60 (0.89–2.88)	43	1.14 (0.75–1.73)
Elementary school or lower	3243	185	1.42 (0.97–2.09)	205	1.31 (0.84–2.04)	68	1.30 (0.71–2.37)	346	1.50 (1.02–2.20)
Occupation									
Non-manual	1328	9	1.00 (reference)	6	1.00 (reference)	3	1.00 (reference)	3	1.00 (reference)
Manual	5010	167	2.25 (1.08–4.70)	102	2.07 (0.82–5.23)	79	6.01 (1.69–21.37)	144	5.31 (1.50–18.84)
Other	3799	149	3.03 (1.40–6.55)	190	3.23 (1.25–8.38)	53	5.50 (1.49–20.39)	314	9.06 (2.54–32.33)
Occupational class									
High/middle-high/middle class	2617	48	1.00 (reference)	18	1.00 (reference)	23	1.00 (reference)	32	1.00 (reference)
Laborers	1467	20	0.69 (0.39–1.21)	17	1.94 (0.95–3.99)	17	1.36 (0.69–2.69)	17	1.13 (0.59–2.19)
Agricultural/fishery/self-employed	799	76	1.56 (0.97–2.50)	44	1.37 (0.71–2.66)	27	1.10 (0.53–2.29)	60	1.04 (0.60–1.78)
Low social class	1509	91	1.47 (0.97–2.23)	82	2.13 (1.13–4.04)	33	1.42 (0.70–2.86)	141	2.10 (1.30–3.39)
Other	3745	90	1.58 (0.96–2.61)	137	2.32 (1.18–4.56)	35	0.91 (0.42–1.94)	211	2.01 (1.19–3.41)
Monthly household income (USD)									
≥3000	925	17	1.00 (reference)	10	1.00 (reference)	1	1.00 (reference)	19	1.00 (reference)
2000–2999	1713	32	1.01 (0.51–1.97)	30	1.67 (0.76–3.69)	14	8.57 (1.11–65.98)	31	1.01 (0.55–1.85)
1000–1999	4019	96	1.26 (0.69–2.30)	66	1.65 (0.78–3.49)	42	9.24 (1.27–67.19)	121	1.38 (0.80–2.35)
<1000	3480	180	1.31 (0.71–2.45)	192	2.33 (1.08–5.03)	78	13.72 (1.90–99.09)	290	1.81 (1.05–3.15)
Equivalized income quintile									
I (highest)	1914	37	1.00 (reference)	24	1.00 (reference)	11	1.00 (reference)	44	1.00 (reference)
II	2128	43	1.09 (0.67–1.79)	36	1.69 (0.95–2.98)	15	1.30 (0.56-3.02)	37	0.70 (0.44–1.09)
III	1965	44	1.13 (0.67–1.88)	38	1.56 (0.88–2.76)	24	1.79 (0.82-3.92)	65	1.09 (0.69–1.73)
IV	2137	74	1.24 (0.79–1.94)	74	2.01 (1.17–3.46)	40	2.50 (1.16-5.36)	110	1.53 (1.04–2.26)
V (lowest)	1993	127	1.37 (0.89–2.10)	126	1.90 (1.13–3.19)	45	1.97 (0.91-4.24)	205	1.51 (1.04–2.20)
Self-rated living standard									
Rich or higher	368	8	1.00 (reference)	11	1.00 (reference)	2	1.00 (reference)	17	1.00 (reference)
Fair	5768	147	1.40 (0.66–2.99)	126	0.87 (0.41–1.86)	67	2.58 (0.56-11.97)	180	0.79 (0.45–1.39)
Poor	3318	135	1.71 (0.79–3.70)	111	1.05 (0.48–2.30)	47	2.12 (0.45-9.98)	203	1.23 (0.70–2.14)
Very poor	683	35	1.87 (0.82–4.28)	50	1.59 (0.71–3.57)	19	5.35 (1.07-26.86)	61	1.39 (0.76–2.55)

*USD* US dollars; *RR* relative risk; *CI* confidence interval

## Discussion

This 12-year follow-up study of mortality in South Korea used a large representative sample to explore the relationship of mortality with a wide range of SEP indicators. The results showed strong evidence that individuals with disadvantaged SEP indicators had greater all-cause mortality risks than their counterparts. However, the magnitude of the relationship between and SEP and mortality varied depending on gender, age, and specific SEP indicators. Our cause-specific analyses using equivalized income quintiles showed that the magnitude of mortality inequalities tended to be greater for cardiovascular disease and external causes than for cancer. Studies on socioeconomic inequalities in mortality using long-term follow-up data from nationally representative samples are rare in Asian countries. Moreover, disparities in mortality associated with a wide range of SEP indicators have not been frequently investigated in Western countries.

A direct comparison of the magnitude of the effects of SEP indicators on mortality found in this study with the findings of studies from other countries would be difficult, since the magnitude can be expected to differ depending on the subjects included in a study, the national representativeness of the sample, the choice and categorization of SEP indicators, and adjusted covariates. A prior study presented inequalities in all-cause mortality in several Asian countries [10], and found that the magnitude of mortality inequalities associated with education, occupation, and income was relatively higher in studies of the Korean population than in other Asian studies [10]. The magnitude of socioeconomic inequalities in all-cause mortality found in this study is slightly lower than what has been observed in prior Korean studies with shorter follow-up periods [21, 23], but generally greater than the average RRs from other Asian countries [10]. A relatively small number of deaths with shorter follow-up periods might have resulted in greater mortality risks in the prior South Korean studies [21, 23]. A recent study using data from the Asia Pacific Cohort Studies Collaboration showed that the average risk of dying among those with primary education or no formal education was 1.64 (95 % CI: 1.46–1.85) times greater than those with tertiary education [11]. When we estimated the RR for those with an elementary or lower level of formal education compared to those with a college or higher level of education, the RR was 1.76 (95 % CI: 1.27–2.43). These international comparative studies [10, 11] and our own estimate indicate that South Korea exhibits similar or relatively greater levels of socioeconomic inequalities in all-cause mortality in comparison to other Asian countries. Economic crises, associated neoliberal structural reforms, and increased income inequality during the past decades might have contributed to the emergence of these dynamics [30]. Several South Korean studies provided evidence that the magnitude of health inequalities in terms of life expectancy and self-rated health became greater after late 1990s’ Asian economic crisis than before [30–32]. However, further nationally representative studies directly comparing the magnitude of socioeconomic inequalities in mortality among Asian countries are warranted.

Education has been the most commonly used SEP indicator in research on socioeconomic disparities in mortality [3, 4, 10, 11, 33, 34]. Occupation (manual vs. non-manual occupations) and income have also often been used to examine mortality differentials [3, 10, 33, 34]. In addition to these conventional SEP indicators, this study employed a wide range of SEP indicators, including occupational class, employment status, type of health insurance, self-rated living standard, living expenditures, home ownership, and housing type, and identified a variety of disparities in mortality associated with SEP indicators. Several previous studies have examined the relative importance of several SEP indicators in predicting future mortality [21, 26, 35]. As seen in Additional file 1: Table S4, all three types of SEP indicators were independently associated with increased risks of mortality after simultaneous adjustment for education, occupation, and equivalized income, which corresponds to the findings of previous studies [21, 26].

The results of our analysis showed that the magnitude of socioeconomic mortality differentials was generally greater in young adults than in elderly people, although SEP-mortality associations were also detected for equivalized income, health insurance, and living standards among those 65 years of age and older. Many international studies have also reported relatively smaller magnitudes of mortality inequalities in the elderly than in young adults [4, 36, 37]. The attenuation of socioeconomic differences in mortality with age has been explained as the result of selective survival, in which persons with low SEP who survive to an advanced age are likely to be very healthy [38, 39].

It has been suggested that the magnitude of socioeconomic inequalities in overall mortality is greater in men than in women [3, 4]. However, gender differences in mortality inequalities may vary depending on SEP indicators, measures of inequality, and causes of death [3, 4, 33, 34, 40, 41]. In this study, the RRs of mortality associated with education tended to be similar or even greater among women than men. Education is measured individually in both men and women, while SEP indicators based on occupation and income usually reflect men’s economic activities outside a household [42]. However, prior unlinked Korean data generally presented greater inequalities in the RR for mortality associated with education in men compared to women [28, 35]. Further studies using individually linked longitudinal data with larger samples are required to determine the relative magnitude of mortality inequalities associated with education in Korean men and women.

Our cause-specific analyses found very high RRs for external causes associated with occupation, monthly household income, and self-rated living standards. However, the use of equivalized income quintiles enhanced the comparability of RRs for four broad causes of death by evenly allocating study subjects into each income quintile, leading to comparable numbers of deaths in each category. Our analysis showed that the relative size of mortality inequalities tended to be greater for cardiovascular disease and external causes of death than for cancer. Many international studies on cause-specific mortality inequalities have provided evidence that mortality inequalities in cancer deaths are relatively smaller than in other causes of death, especially among women [3, 4, 9].

This study has strengths and limitations. It was based on follow-up mortality data from a nationally representative sample and thus captured the national status of mortality inequalities in South Korea. Using 12-year mortality follow-up data, we presented mortality differentials according to gender and cause of death, which have not been reported in previous studies using the KNHANES [21, 23, 24]. In addition, we employed a wide range of SEP indicators and found higher risks of mortality in socioeconomically vulnerable groups in Korean society. However, the numbers of deaths and subjects were not enough to determine patterns in inequalities in mortality from even more specific causes of death.

## Conclusions

In summary, we presented mortality inequalities associated with a wide range of SEP indicators using nationally representative follow-up mortality data from the South Korean population. We also identified cause-, gender-, and age-specific patterns of mortality inequalities. We found that inequalities in mortality were associated with several SEP indicators, gender, and age, and that the magnitude of these inequalities differed depending on the broad cause of death. Study results from this study as well as prior Asian studies [10–17] provide evidence that socioeconomic inequalities in mortality, which have been commonly reported in the West, also exist in Asian countries. More rigorous comparative studies among Asian countries will allow us to develop a more specific explanation on the international differences in the magnitude of mortality inequalities. In addition, the South Korean economic development, previously described as effective in both economic growth and relatively equitable income distribution [43, 44], should be scrutinized regarding its impact on socioeconomic mortality inequalities. This is especially important given that the generalizability of the South Korea’s economic development model to developing countries has been suggested [45–47]. Moreover, policy measures to reduce inequalities in mortality should be implemented in South Korea.

## Additional file

Additional file 1: Table S1. Numbers of study subjects and deaths, follow-up duration (person-years), and 12-year mortality rates by gender and age groups in subjects of the 1998 and 2001 Korea National Health and Nutrition Examination Surveys (KNHANES). **Table S2.** Age group-specific relative risks (adjusted for age and gender) of mortality from all causes according to socioeconomic position indicators: follow-up 12-year mortality data from the 1998 and 2001 Korea National Health and Nutrition Examination Surveys. **Table S3.** Gender-specific relative risks (adjusted for age) of mortality from all causes of death according to socioeconomic position indicators: follow-up 12-year mortality data from the 1998 and 2001 Korea National Health and Nutrition Examination Surveys. **Table S4.** Age- and gender-adjusted relative risks of all-cause mortality in a model simultaneously adjusting for education, occupation, and monthly household income. (DOCX 44 kb)

## Abbreviations

CI: confidence intervals
ICD-10: International Classification of Disease, 10th Revision
KNHANES: Korean National Health and Nutrition Examination Survey
RR: relative risk
SEP: socioeconomic position
USD: US dollar
