# Supplementary material for: Socioeconomic Inequality in mortality using 12-year follow-up data from nationally representative surveys in South Korea
Source: Int J Equity Health. 2016 Mar 22;15:51. doi: 10.1186/s12939-016-0341-9 (PMC4802872; doi:10.1186/s12939-016-0341-9)
Supplement: Additional file 1: Table S1. — Numbers of study subjects and deaths, follow-up duration (person-years), and 12-year mortality rates by gender and age groups in subjects of the 1998 and 2001 Korea National Health and Nutrition Examination Surveys (KNHANES). Table S2. Age group-specific relative risks (adjusted for age and gender) of mortality from all causes according to socioeconomic position indicators: follow-up 12-year mortality data from the 1998 and 2001 Korea National Health and Nutrition Examination Surveys. Table S3. Gender-specific relative risks (adjusted for age) of mortality from all causes of death according to socioeconomic position indicators: follow-up 12-year mortality data from the 1998 and 2001 Korea National Health and Nutrition Examination Surveys. Table S4. Age- and gender-adjusted relative risks of all-cause mortality in a model simultaneously adjusting for education, occupation, and monthly household income. (DOCX 44 kb) [file 12939_2016_341_MOESM1_ESM.docx]

**Additional File 1**

Table S1. Numbers of study subjects and deaths, follow-up duration (person-years), and 12-year mortality rates by gender and age groups in subjects of the 1998 and 2001 Korea National Health and Nutrition Examination Surveys (KNHANES).

| Gender/age groups (years) | 1998 KNHANES data | | | |  | 2001 KNHANES data | | | |
| --- | --- | --- | --- | --- | --- | --- | --- | --- | --- |
|  | Number of subjects | Follow-up duration (PY) | Number of deaths | Mortality rates (per 1000 PY) |  | Number of subjects | Follow-up duration (PY) | Number of deaths | Mortality rates (per 1000 PY) |
| Men |  |  |  |  |  |  |  |  |  |
| 30–39 | 824 | 11538 | 17 | 1.5 |  | 643 | 7162 | 5 | 0.7 |
| 40–49 | 702 | 9549 | 62 | 6.5 |  | 596 | 6586 | 19 | 2.9 |
| 50–59 | 509 | 6611 | 97 | 14.7 |  | 371 | 3989 | 36 | 9.0 |
| 60–69 | 389 | 4457 | 161 | 36.1 |  | 296 | 2945 | 77 | 26.1 |
| 70–79 | 167 | 1557 | 116 | 74.5 |  | 131 | 1141 | 72 | 63.1 |
| ≥80 | 24 | 154 | 23 | 149.1 |  | 30 | 140 | 26 | 185.7 |
| Subtotal | 2615 | 33866 | 476 | 14.1 |  | 2067 | 21963 | 235 | 10.7 |
| Women |  |  |  |  |  |  |  |  |  |
| 30–39 | 917 | 12907 | 17 | 1.3 |  | 805 | 8967 | 8 | 0.9 |
| 40–49 | 720 | 10058 | 18 | 1.8 |  | 680 | 7561 | 8 | 1.1 |
| 50–59 | 554 | 7657 | 31 | 4.0 |  | 396 | 4344 | 21 | 4.8 |
| 60–69 | 458 | 6026 | 77 | 12.8 |  | 346 | 3685 | 40 | 10.9 |
| 70–79 | 245 | 2780 | 119 | 42.8 |  | 203 | 1950 | 62 | 31.8 |
| ≥80 | 71 | 450 | 62 | 137.8 |  | 60 | 396 | 45 | 113.6 |
| Subtotal | 2965 | 39878 | 324 | 8.1 |  | 2490 | 26903 | 184 | 6.8 |
| Total | 5580 | 73744 | 800 | 10.8 |  | 4557 | 48866 | 419 | 8.6 |

PY, person-years.

Table S2. Age group-specific relative risks (adjusted for age and gender) of mortality from all causes according to socioeconomic position indicators: follow-up 12-year mortality data from the 1998 and 2001 Korea National Health and Nutrition Examination Surveys.

|  | Men and women 30 years of age and older | |  | Men and women 30–64 years of age | |  | Men and women 65 years of age and older | |
| --- | --- | --- | --- | --- | --- | --- | --- | --- |
|  | Number of subjects (deaths) | RR (95% CI) |  | Number of subjects (deaths) | RR (95% CI) |  | Number of subjects (deaths) | RR (95% CI) |
| Education |  |  |  |  |  |  |  |  |
| High school or higher | 5269 (258) | 1.00 (reference) |  | 5074 (183) | 1.00 (reference) |  | 195 (75) | 1.00 (reference) |
| Middle school | 1625 (157) | 1.25 (1.00–1.57) |  | 1503 (103) | 1.35 (1.02–1.79) |  | 122 (54) | 1.11 (0.75–1.65) |
| Elementary school or lower | 3243 (804) | 1.40 (1.13–1.74) |  | 1994 (220) | 1.56 (1.16–2.11) |  | 1249 (584) | 1.21 (0.90–1.62) |
| Education |  |  |  |  |  |  |  |  |
| Middle school or higher | 6894 (415) | 1.00 (reference) |  | 6577 (286) | 1.00 (reference) |  | 317 (129} | 1.00 (reference) |
| Elementary school | 2031 (344) | 1.20 (0.99–1.44) |  | 1570 (169) | 1.33 (1.02–1.73) |  | 461 (175) | 1.05 (0.81–1.36) |
| No formal education | 1212 (460) | 1.52 (1.21–1.92) |  | 424 (51) | 1.69 (1.08–2.63) |  | 788 (409) | 1.33 (1.00–1.75) |
| Employment status |  |  |  |  |  |  |  |  |
| Employed, standard | 2210 (65) | 1.00 (reference) |  | 2184 (61) | 1.00 (reference) |  | 26 (4) | 1.00 (reference) |
| Self-employed | 2018 (300) | 1.65 (1.21–2.24) |  | 1708 (163) | 1.68 (1.20–2.35) |  | 310 (137) | 2.93 (1.02–8.36) |
| Employers | 363 (18) | 1.18 (0.68–2.04) |  | 355 (15) | 1.05 (0.58–1.91) |  | 8 (3) | 2.84 (0.67–12.04) |
| Employed, non-standard | 799 (73) | 2.30 (1.55–3.41) |  | 745 (55) | 2.59 (1.71–3.91) |  | 54 (18) | 2.86 (0.87-9.40) |
| Other | 4747 (763) | 2.28 (1.68–3.09) |  | 3579 (212) | 2.23 (1.57–3.18) |  | 1168 (551) | 4.37 (1.55–12.35) |
| Occupational class |  |  |  |  |  |  |  |  |
| High/middle-high/middle class | 2617 (121) | 1.00 (reference) |  | 2546 (96) | 1.00 (reference) |  | 71 (25) | 1.00 (reference) |
| Laborers | 1467 (71) | 1.12 (0.81–1.55) |  | 1442 (65) | 1.33 (0.95–1.87) |  | 25 (6) | 0.39 (0.14–1.09) |
| Agricultural/fishery/self-employed | 799 (207) | 1.17 (0.88–1.55) |  | 553 (92) | 1.41 (0.97–2.04) |  | 246 (115) | 0.84 (0.51–1.38) |
| Low social class | 1509 (347) | 1.69 (1.31–2.18) |  | 1048 (140) | 2.08 (1.52–2.84) |  | 461 (207) | 1.18 (0.73–1.90) |
| Other | 3745 (473) | 1.67 (1.26–2.22) |  | 2982 (113) | 1.79 (1.18–2.71) |  | 763 (360) | 1.27 (0.77–2.09) |
| Type of health insurance |  |  |  |  |  |  |  |  |
| Public servant health insurance | 955 (117) | 1.00 (reference) |  | 784 (36) | 1.00 (reference) |  | 171 (81) | 1.00 (reference) |
| Employee health insurance | 3633 (370) | 1.09 (0.85–1.38) |  | 3112 (162) | 1.33 (0.88–2.02) |  | 521 (208) | 0.92 (0.68–1.25) |
| Self-employed health insurance | 5164 (610) | 1.25 (1.00–1.57) |  | 4443 (277) | 1.46 (0.98–2.16) |  | 721 (333) | 1.08 (0.82–1.44) |
| Medical aid program | 315 (108) | 1.83 (1.33–2.52) |  | 170 (23) | 3.40 (1.84–6.27) |  | 145 (85) | 1.39 (0.97–1.99) |
| No health insurance | 70 (14) | 2.85 (1.54–5.28) |  | 62 (8) | 3.01 (1.26–7.19) |  | 8 (6) | 3.04 (1.20–7.73) |
| Monthly living expenditures, USD (2001 data) | |  |  |  |  |  |  |  |
| ≥2000 | 912 (50) | 1.00 (reference) |  | 822 (17) | 1.00 (reference) |  | 90 (33) | 1.00 (reference) |
| 1500–1999 | 836 (43) | 1.65 (0.97–2.82) |  | 783 (18) | 1.80 (0.77–4.20) |  | 53 (25) | 1.54 (0.82–2.91) |
| 1000–1499 | 1276 (88) | 1.43 (0.92–2.20) |  | 1150 (43) | 1.69 (0.87–3.30) |  | 126 (45) | 1.28 (0.72–2.26) |
| 500–999 | 1083 (126) | 1.46 (0.95–2.25) |  | 866 (47) | 1.97 (0.99–3.91) |  | 217 (79) | 1.23 (0.70–2.16) |
| <500 | 450 (112) | 1.71 (1.09–2.66) |  | 226 (16) | 2.43 (1.04–5.67) |  | 224 (96) | 1.44 (0.83–2.50) |
| Home ownership (2001 data) |  |  |  |  |  |  |  |  |
| Owned home | 3187 (331) | 1.00 (reference) |  | 2607 (104) | 1.00 (reference) |  | 580 (227) | 1.00 (reference) |
| Did not own home | 1370 (88) | 1.25 (0.93–1.67) |  | 1240 (37) | 1.27 (0.81–2.00) |  | 130 (51) | 1.24 (0.85–1.81) |
| Housing type (2001 data) |  |  |  |  |  |  |  |  |
| Apartment | 1694 (86) | 1.00 (reference) |  | 1540 (39) | 1.00 (reference) |  | 154 (47) | 1.00 (reference) |
| House | 2138 (268) | 1.65 (0.86–3.16) |  | 1685 (82) | 1.46 (0.55–3.89) |  | 453 (186) | 1.50 (0.72–3.11) |
| Other | 725 (65) | 1.61 (0.80–3.22) |  | 622 (20) | 0.98 (0.33–2.89) |  | 103 (45) | 1.82 (0.80–4.13) |

RR, relative risk; CI, confidence interval; USD, US dollars.

Table S3. Gender-specific relative risks (adjusted for age) of mortality from all causes of death according to socioeconomic position indicators: follow-up 12-year mortality data from the 1998 and 2001 Korea National Health and Nutrition Examination Surveys.

|  | Men 30 years of age and older | |  | Women 30 years of age and older | |
| --- | --- | --- | --- | --- | --- |
|  | Number of subjects (deaths) | RR (95% CI) |  | Number of subjects (deaths) | RR (95% CI) |
| Education |  |  |  |  |  |
| High school or higher | 2885 (218) | 1.00 (reference) |  | 2384 (40) | 1.00 (reference) |
| Middle school | 746 (122) | 1.25 (0.98–1.60) |  | 879 (35) | 1.40 (0.81–2.43) |
| Elementary school or lower | 1051 (371) | 1.34 (1.06–1.69) |  | 2192 (433) | 1.89 (1.09–3.26) |
| Education |  |  |  |  |  |
| Middle school or higher | 3631 (340} | 1.00 (reference) |  | 3263 (75) | 1.00 (reference) |
| Elementary school | 805 (228) | 1.18 (0.95–1.46) |  | 1226 (116) | 1.41 (0.91–2.18) |
| No formal education | 246 (143) | 1.39 (1.03–1.89) |  | 966 (317) | 2.01 (1.26–3.21) |
| Employment status |  |  |  |  |  |
| Employed, standard | 1629 (57) | 1.00 (reference) |  | 581 (8) | 1.00 (reference) |
| Self-employed | 1525 (261) | 1.64 (1.18–2.29) |  | 493 (39) | 1.72 (0.76–3.89) |
| Employers | 300 (18) | 1.23 (0.70–2.15) |  | 63 (0) | NA |
| Employed, non-standard | 365 (54) | 2.45 (1.58–3.82) |  | 434 (19) | 1.81 (0.72–4.52) |
| Other | 863 (321) | 2.48 (1.77–3.48) |  | 3884 (442) | 2.09 (0.97–4.47) |
| Occupational class |  |  |  |  |  |
| High/middle-high/middle class | 1962 (104) | 1.00 (reference) |  | 655 (17) | 1.00 (reference) |
| Laborers | 938 (60) | 1.15 (0.81–1.63) |  | 529 (11) | 0.88 (0.39–1.98) |
| Agricultural/fishery/self-employed | 656 (183) | 1.25 (0.91–1.73) |  | 143 (24) | 0.92 (0.47–1.82) |
| Low social class | 919 (269) | 1.90 (1.43–2.52) |  | 590 (78) | 1.21 (0.66–2.20) |
| Other | 207 (95) | 2.10 (1.44–3.07) |  | 3538 (378) | 1.23 (0.71–2.13) |
| Type of health insurance |  |  |  |  |  |
| Public servant health insurance | 451 (66) | 1.00 (reference) |  | 504 (51) | 1.00 (reference) |
| Employee health insurance | 1706 (229) | 1.32 (0.98–1.77) |  | 1927 (141) | 0.79 (0.54–1.16) |
| Self-employed health insurance | 2390 (368) | 1.49 (1.13–1.98) |  | 2774 (242) | 0.90 (0.63–1.29) |
| Medical aid program | 101 (40) | 1.75 (1.09–2.81) |  | 214 (68) | 1.59 (1.03–2.46) |
| No health insurance | 34 (8) | 3.53 (1.50–8.34) |  | 36 (6) | 2.14 (0.92–4.99) |
| Monthly living expenditures, USD (2001 data) | |  |  |  |  |
| ≥2000 | 412 (24) | 1.00 (reference) |  | 500 (26) | 1.00 (reference) |
| 1500–1999 | 389 (27) | 2.17 (1.08–4.38) |  | 447 (16) | 1.08 (0.45–2.58) |
| 1000–1499 | 604 (52) | 1.95 (1.10–3.45) |  | 672 (36) | 0.96 (0.48–1.95) |
| 500–999 | 503 (84) | 2.11 (1.22–3.65) |  | 580 (42) | 0.97 (0.47–1.98) |
| <500 | 159 (48) | 2.16 (1.18–3.97) |  | 291 (64) | 1.41 (0.69–2.88) |
| Home ownership (2001 data) |  |  |  |  |  |
| Owned home | 1442 (192) | 1.00 (reference) |  | 1745 (139) | 1.00 (reference) |
| Did not own home | 625 (43) | 1.03 (0.69–1.55) |  | 745 (45) | 1.56 (0.97–2.51) |
| Housing types (2001 data) |  |  |  |  |  |
| Apartment | 764 (46) | 1.00 (reference) |  | 930 (40) | 1.00 (reference) |
| House | 960 (147) | 1.28 (0.54–3.05) |  | 1178 (121) | 2.15 (0.87–5.31) |
| Other | 343 (42) | 1.10 (0.45–2.68) |  | 382 (23) | 2.35 (0.81–6.82) |

RR, relative risk; CI, confidence interval; USD, US dollars.

Table S4. Age- and gender-adjusted relative risks of all-cause mortality in a model simultaneously adjusting for education, occupation, and monthly household income.

|  | RR (95% CI) |
| --- | --- |
| Education |  |
| College or higher | 1.00 (reference) |
| High school | 1.05 (0.77–1.43) |
| Middle school | 1.20 (0.87–1.68) |
| Elementary school | 1.23 (0.88–1.72) |
| No formal education | 1.62 (1.12–2.35) |
| Occupation |  |
| Non-manual | 1.00 (reference) |
| Manual | 2.58 (1.54–4.33) |
| Other | 3.74 (2.20–6.35) |
| Monthly household income (USD) |  |
| ≥3000 | 1.00 (reference) |
| 2000–2999 | 1.17 (0.80–1.70) |
| 1000–1999 | 1.29 (0.91–1.83) |
| <1000 | 1.46 (1.01–2.10) |

RR, relative risk; CI, confidence interval; USD, US dollars.
